# Increasing delivery of an outdoor journey intervention to people with stroke: A feasibility study involving five community rehabilitation teams

**DOI:** 10.1186/1748-5908-5-59

**Published:** 2010-07-29

**Authors:** Annie McCluskey, Sandy Middleton

**Affiliations:** 1 Community-Based Health Care Research Unit, Faculty of Health Sciences, The University of Sydney, New South Wales, Australia; 2 Royal Rehabilitation Centre Sydney, New South Wales, Australia; 3 Nursing Research Institute, St Vincent's and Mater Health Sydney and the Australian Catholic University, New South Wales, Australia; 4 National Centre for Clinical Outcomes Research (NaCCOR), Nursing and Midwifery, The Australian Catholic University, Australia

## Abstract

**Background:**

Contrary to recommendations in a national clinical guideline, baseline audits from five community-based stroke rehabilitation teams demonstrated an evidence-practice gap; only 17% of eligible people with stroke were receiving targeted rehabilitation by occupational therapists and physiotherapists to increase outdoor journeys. The primary aim of this feasibility study was to design, test, and evaluate the impact of an implementation program intended to change the behaviour of community rehabilitation teams. A secondary aim was to measure the impact of this change on client outcomes.

**Methods:**

A before-and-after study design was used. The primary data collection method was a medical record audit. Five community rehabilitation teams and a total of 12 professionals were recruited, including occupational therapists, physiotherapists, and a therapy assistant. A medical record audit was conducted twice over 12 months (total of 77 records pre-intervention, 53 records post-intervention) against a guideline recommendation about delivering outdoor journey sessions to people with stroke. A behavioural intervention (the 'Out-and-About Implementation Program') was used to help change team practice. Active components of the intervention included feedback about the audit, barrier identification, and tailored education to target known barriers. The primary outcome measure was the proportion of medical records containing evidence of multiple outdoor journey sessions. Other outcomes of interest included the proportion of medical records that contained evidence of screening for outdoor journeys and driving by team members, and changes in patient outcomes. A small sample of community-dwelling people with stroke (n = 23) provided pre-post outcome data over three months. Data were analysed using descriptive statistics and t-tests.

**Results:**

Medical record audits found that teams were delivering six or more outdoor journeys to 17% of people with stroke pre-intervention, rising to 32% by 12 months post-intervention. This change represents a modest increase in practice behaviour (15%) across teams. More people with stroke (57%) reported getting out of the house as often as they wanted after receiving the outdoor journey intervention compared to 35% one year earlier; other quality of life outcomes also improved.

**Conclusions:**

The 'Out-and-About Implementation Program' helped rehabilitation teams to change their practice, implement evidence, and improve client outcomes. This behavioural intervention requires more rigorous evaluation using a cluster randomised trial design.

## Background

Over 60,000 Australians experience a stroke each year [1]. Less than 10% of people with stroke can walk fast enough to cross a road safely when they leave hospital [2]. Up to 50% fall at home in the first six months after discharge [3]. Two-thirds of people are never able to resume driving after a stroke [4,5], and many cannot use public transport. Unless community rehabilitation is provided, many people with stroke will experience social isolation, reduced physical activity, and poor quality of life.

### Evidence-based community stroke rehabilitation

Community rehabilitation, including mobility and transport training, can improve health outcomes for people with stroke [6-8]. A systematic review of 21 trials of physiotherapy exercise programs for people with stroke reported gains in walking speed and distance following task-specific training [6]. One of these trials reported increased walking capacity following four weeks of treadmill training and overground walking practice in community-dwelling stroke survivors with speed gains being maintained after three months [8].

Yet, people with stroke who received several weeks of community mobility training report a lack of confidence negotiating ramps, escalators, and shopping malls [9]. Further, repeated practice walking indoors in a hospital gym did not automatically lead to improved walking outdoors. To gain confidence and skills, people with stroke seem to need multiple escorted journeys in their local community with a rehabilitation therapist.

Increased outdoor journeys and quality of life post-stroke were the focus of one trial conducted in England [7]. This trial compared the distribution of leaflets describing local transport options (control group), with the same leaflets plus delivery of up to seven individual sessions over a three-month period by occupational therapists who encouraged outdoor mobility and travel (intervention group). Participants in the intervention group were escorted by therapists on walks, bus, and taxi trips until they felt confident to go out alone [10]. Therapists also provided transport information to the intervention group. After four months and a median of six sessions, twice as many people from the intervention group reported getting out as often as they wanted (RR 1.72, 95% CI 1.25 to 3.27) [7]. Between-group differences were maintained at 10 months, long after therapy had ceased.

### The evidence-practice gap

Australian national stroke guidelines recommend escorted journeys, written transport information, and ambulation training following stroke [11]. These recommendations are consistent with findings from the randomised trial by Logan and colleagues [7,10]. However, anecdotally, a large evidence-practice gap appeared to exist in local community stroke rehabilitation practice in our region.

Barriers to translating evidence into practice include lack of knowledge about the evidence, limited skills and competence, and consumer expectations about therapy [12,13]. Implementation programs use a number of 'interventions' to target local barriers and change practice [14,15]. These interventions include dissemination of clinical guidelines and other educational materials [16], education meetings, audit and performance feedback [17], reminder systems, and a combination of these. The efficacy of implementation interventions was evaluated in a systematic review that included 235 studies [18,19]; in that review, most interventions led to small changes in practice of up to 10%. Larger changes can be expected when compliance with best practice is low at baseline. We used this 'evidence about getting evidence into practice' to design and test an implementation program.

The primary aim of the present study was to design, pilot test, and evaluate the impact of an implementation program intended to change the behaviour of community rehabilitation teams. The behaviour measured was delivery of multiple outdoor journey sessions to people with stroke, consistent with a national guideline recommendation. A secondary aim was to evaluate the impact of practice change on client outcomes.

## Methods

A before-and-after design was used. The primary data collection method was medical record audit, conducted on two cohorts: a pre-intervention cohort, and another different cohort 12 months later. A secondary data collection method was administration of standardised outcome measures to people with stroke who received the outdoor journey intervention.

### The Sample

#### Rehabilitation team participants

A purposive sample of five community rehabilitation teams was recruited in Sydney, Australia representing different models of service delivery (out-patient, domiciliary, and day hospital). To be eligible, teams had to employ at least one occupational therapist and one physiotherapist, and have seen at least ten people with stroke in the previous six months. These professionals helped conduct medical records audits, received feedback from the audits, were interviewed about barriers to implementation, attended an education session, and delivered the outdoor journey sessions to people with stroke on their caseload.

#### Participants with stroke

Therapists from two teams consented to their clients being recruited. Funding did not permit data collection across all five teams. Community-dwelling people with stroke seen by two participating teams were invited to participate in the study if they met the following criteria: they needed rehabilitation to increase their outdoor journeys (based on screening questions asked by a team member); they agreed to participate in multiple outdoor journey sessions; and they agreed to be interviewed by AM and provide additional outcome data.

#### The out-and-about implementation program

The intervention provided to help rehabilitation therapists implement the outdoor journeys was named the 'Out-and-About Implementation Program'. The program aimed to change practice and included three active components: medical record audits followed by feedback, barrier identification, and education to target known local barriers.

Medical record audits were conducted retrospectively by AM and two professionals from each team. We requested 100 consecutive records (20 records for each of the five teams) of people with stroke who had received therapy (for any reason) in the previous 12 months from a team occupational therapist, physiotherapist, or both. One exception was a new team that had been established six months earlier, and had only seen 10 people with stroke. In that case, we requested all of their records for people with stroke seen since service commencement. Multiple auditors were used to raise professionals' awareness of their practice, and the practice of their team, by engaging them in audits. Each professional audited at least three medical records. Two medical files from the total sample were double coded by the first investigator to check for consistency. Differences were discussed and consensus reached when necessary. No formal study of rater agreement was conducted.

Audit criteria were rated using yes/no response options. Questions were asked about screening and assessments conducted, intervention provided, goals set and outcomes measured in relation to transport, outdoor mobility, and outings. Any occasions of service that focussed on improving outdoor journeys were counted. A written summary of each team's performance was provided to teams within eight weeks by AM.

Feedback of results from the first audit was provided to each team about their compliance with key criteria, with comparison to the overall compliance by the five teams. Each team then set targets for the next 12 months (*e.g.*, '50% of people with stroke will have written evidence that driving has been discussed').

A second retrospective audit of medical records was conducted 12 months later using identical tools and processes to the first audit. Medical files were requested of 100 people with stroke treated after the half-day implementation training workshop (20 consecutive records for each of the five teams). Nine rehabilitation professionals audited the medical records in addition to AM.

Barrier identification was conducted concurrently with the audit process. To identify barriers, we used two methods that have been recommended for implementation research [12]. First, we conducted in-depth interviews (described elsewhere [20]) with allied health professionals from two teams, and then transcribed and analysed the content. Interviewees were asked to describe what they knew about the outdoor journey intervention, including the published evidence, and factors that might help or hinder their team from implementing the outdoor journey intervention. Prompt questions were used to enquire about skills and knowledge, staffing, resources, assessment procedures, screening and report-writing systems, and treatment routines. Findings were then used to inform the content of a workshop.

#### Education

A half-day workshop was run in August 2007. The workshop was lead by AM. First, we presented a critical appraisal of the original randomised trial by Logan and colleagues [7], and a description of the complex outdoor journey intervention [10]. Therapists were alerted to the national clinical guideline recommendation about the intervention [11].

Second, baseline audit data were presented with the permission of the five teams. Based on the review by Grimshaw and colleagues [19], consensus was reached at the workshop that a 10% improvement in the target practice behaviours would be the goal for teams following the implementation program (*i.e.*, the pre-determined minimum clinically worthwhile difference).

Third, a written document was presented and discussed ('Increasing outdoor journeys after stroke: Protocols for use by rehabilitation professionals'). Protocols were provided for upgrading walking, bus and train travel training, trialling motorised scooters, addressing return to driving, and providing written information about transport options. These protocols had been prepared by the AM with advice from local team members.

Fourth, two case studies were presented by occupational therapists who had delivered escorted journeys to people with stroke. Each case study included goals of the person with stroke, treatment progression, and safety tips. A videotaped interview was also presented showing a person with stroke who described the benefits of being assisted to get out of the house. Participants then practiced writing sample goals related to outdoor journeys and community participation.

Finally, potential barriers and enablers to delivering the outdoor journeys were identified, then discussed by workshop participants in pairs or teams. Examples and quotes were presented from the earlier in-depth interviews conducted with team members [20]. Participants identified strengths, weaknesses, opportunities, and threats affecting their team's ability to provide the evidence-based outdoor journey intervention. Solutions were proposed, discussed and documented by team leaders.

### Outcome measures

#### Team outcomes

The primary outcome of team behaviour change was the proportion of people with stroke who received six or more outdoor journey sessions from an occupational therapist, physiotherapist, or therapy assistant. These outcomes were obtained from the same medical record audits that were used to provide feedback to participating teams. Records were requested of consecutive people with stroke seen by teams for 12 months before (pre-intervention) and 12 months after the implementation (post-intervention) training workshop. Secondary outcomes, also obtained from medical record audits, included the proportion of people with stroke who were screened and asked questions about outings, their preferred destinations and modes of travel, and driving status.

#### Patient outcomes

Consecutive people with stroke from two teams who received the outdoor journey intervention and provided consent were visited at home by AM. They were visited on two occasions, once before therapy sessions commenced (baseline) and then again three months later (follow-up). Participants were asked a single question, which was the primary outcome of interest: 'Are you getting out of the house as often as you would like?' (yes/no). Four standardised measures were also completed with assistance from AM, partly to identify a suitable primary outcome measure for a future trial. First, participants completed the Nottingham Extended Activities of Daily Living (NEADL) scale [21], which is a self-report measure comprising 22 questions about community and home-based activities (maximum score 66). The Life Space Assessment (LSA) [22] was also used; this self-report measure records how far a person has walked or travelled in the past month (maximum score 120). The Falls Efficacy Scale (International, FES-I) [23] enquired about concerns regarding the possibility of falling when performing, or thinking about performing, various activities (maximum score 64). The Reintegration to Normal Living Index (RNLI) [24] then measured how well a participant felt they had resumed community-based activities (maximum score 22).

Finally, a list was generated of outings and outdoor journeys completed over the previous seven days, supervised or unsupervised, on foot or in a vehicle. An outing was defined as an excursion into the community beyond the front gate. An outdoor journey was defined as any excursion beyond the front or back door of the house, and included short walks to the post-box or around the garden. An excursion involving a walk to the car, then a car journey to the shops, then a walk into a shopping mall represented one outing but three outdoor journeys. This method of recording outings and outdoor journeys was replicated from the original trial by Logan and colleagues [7].

#### Ethical approval

Ethical approval for the study was obtained from the local area health service (Ref No. 2007/019) and university ethics committee (Ref No. 10092).

#### Sample size

While therapists agreed on a 10% improvement for the target practice behaviour [18,19], the proportion of people with stroke who received six or more outdoor journey sessions, our study was not powered to detect this difference. This would have required recruitment of many more teams, and was beyond the scope of this pilot study that aimed to test the feasibility of the implementation program.

#### Data analysis

Team and patient outcome data were analysed using descriptive statistics including proportions, means/standard deviations, or median/interquartile range. For categorical data and proportions, we used McNemar's repeated measures chi-square test to compare within-group differences. Mean within-group differences were calculated using paired t-tests and 95% confidence intervals for continuous data (NEADL, LSA, FES-I and HADS).

## Results

### Sample characteristics

#### Rehabilitation team participant characteristics

Of the 12 rehabilitation therapists who helped conduct the audits, all except one were female, and all were either an occupational therapist (n = 8) or a physiotherapist (n = 4).

#### Patient participant characteristics

For the pre-intervention cohort of people with stroke (n = 77), the median age was 67.5 years (IQR 54.8 to 77.8); this cohort were a median of 23.5 days post-discharge from hospital or days since referral to the team (IQR 11.0 to 58.8). For the post-intervention cohort (n = 53), the median age was 66.5 years (IQR 50.6 to 75.7); this cohort were a median of 21.5 days post-discharge from hospital or days since referral to the team (IQR 8.0 to 41.6).

#### Medical record audit data

Pre-intervention, 77 of the 100 medical records requested were available for auditing. A year later, when another 100 consecutive records were requested, we located and audited 53 medical records. Some medical records did not contain therapists' notes, while other records were not available for audit. Table 1 presents a summary of audit criteria and the proportion of medical records that complied with each criterion across teams.

**Table 1:** Audit data from medical records across five teams at baseline and follow-up 12 months later

Criteria	% Compliance				% Change
	Baseline		Follow-up		
	(N = 77)		(N = 53)		
**Intervention**: Is there written evidence of intervention aimed at increasing outdoor journeys	*n*	*%*	*n*	*%*	
Six sessions or more	13	17%	17	32%	+15%
Four sessions or more	16	21%	19	39%	+18%
Two sessions or more	27	35%	25	51%	+16%
At least one session	44	57%	37	76%	-19%
No sessions provided	33	43%	16	13%	-30%
**Screening Questions**: Were the following content areas documented?	*n*	*%*	*n*	*%*	
Mobility status	77	100%	53	100%	0.0%
Home access	69	90%	47	89%	-1.0%
Pre-stroke driving status	37	48%	38	72%	+24%
Preferred destinations	19	25%	24	45%	+20%
Preferred modes of travel	27	35%	34	61%	+26%
Reasons for limited outings	26	34%	21	40%	+6.0%
Current outings discussed	39	51%	35	66%	+15%
Number of weekly outings estimated	11	14%	16	30%	+16%

Medical records were audited across five community rehabilitation teams

At the 12-month audit, several notable changes in practice were recorded (≥ 10% change) including better recording and more frequent screening of people with stroke about their driving status (+ 24%), noting of: preferred modes of travel (+ 26%) and weekly outings (+ 15%). The post-intervention audit also revealed better recording and more frequent delivery of outdoor journey sessions (19% more people received one session; 15% more people received six sessions). A greater proportion of people with stroke (76%) received at least one outdoor journey session compared to pre-intervention (57%).

Audit data revealed a modest change in practice across teams, although this difference was not statistically significant. Nearly one-third of people with stroke (32%) received six or more sessions after one year, compared to 17% at baseline (a 15% change). However, there were marked differences between teams (see Table 2). Team four achieved the greatest change in practice (a 34% change). Initially, 36% of people with stroke whose records were audited received six or more outdoor journey sessions. One year later, this proportion had increased to 70% for team four.

**Table 2:** Proportion of medical records audited where people with stroke received six or more outdoor journey sessions (n*, %)

Team	Time of Audit			
	Pre-Intervention (2006 to 2007)		Post-Intervention (2007 to 2008)	
	n *	%	n *	%
Team one	4/22	18.2	2/19	10.5
Team two	3/21	14.3	6/15	40.0
Team three	2/13	15.4	2/7	28.6
Team four	4/11	36.4	7/10	70.0
Team five	0/10	0.0	0/2	0.0
Total	13/77	16.9	17/53	32.1

* *'n' *refers to the number of audited files that contained evidence of outdoor journey sessions, divided by the total number of files audited per team

#### Number of outdoor journey sessions

**Figure 1:**
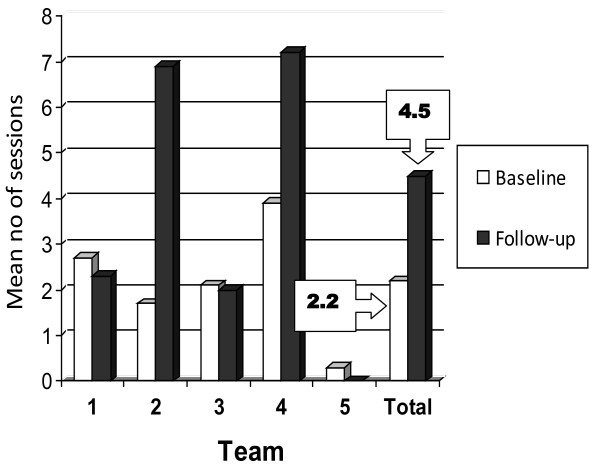
Mean number of outdoor journey sessions delivered by the five community teams as documented in medical records at baseline and follow-up

The number of sessions per person increased from a mean of 2.2 (SD 3.2) at baseline to 4.5 (SD 7.9) after 12 months (median 1.0, IQR 0.0 to 3.0, to median of 2.0, IQR 0.0 to 7.0) (Figure 1). Team four successfully delivered a mean of 7.0 sessions (SD 4.3). Although team two increased the mean number of sessions, their follow-up data were skewed by one person with stroke who received 52 sessions. When that outlier was removed from analysis, the follow-up mean for that team decreased to 3.7 sessions (SD 4.3).

#### Patient outcomes

Outcome data were collected from 23 people with stroke who received outdoor journey sessions from two of the participating teams (see Table 3). The mean age of the sample was 66.7 (SD 12.8), one-half were female (n = 10, 56.5%), and two-thirds drove a car pre-stroke (n = 15, 65.2%). Median time to baseline data collection and commencement of the outdoor journey intervention was 58 days post-stroke (IQR 49 to 111), and 21 days post-discharge (IQR 7 to 40). Only one-third of the sample (34.8%) said that they were getting out as often as they wanted before the outdoor journey sessions began.

**Table 3:** Within-group differences^# ^after three months for people with stroke who received the outdoor journey intervention (Mean/SD) and provided pre-post data (n = 21)

Measure	Pre-test	Post-test	Diff	95% CI	P value
'Are you getting out of the house as often as you want?' (% Yes)	34.8% (n = 8)	57.1% (n = 12)	22.3%	NA	0.219
Number of outdoor journeys† per week	28.2 (18.2)	30.4 (14.3)	2.2	-9.6 to 5.3	0.548
NEADL (0-66)	26.9 (12.6)	35.1 (13.5)	7.3	1.2 to 13.5	0.022 *
RNLI (0-22)	13.9 (5.0)	15.8 (3.1)	1.9	-4.2 to 0.4	0.102
LSA (0-120)	36.4 (13.8)	40.7 (15.2)	4.3	-12.9 to 4.4	0.314
FES-I (0-64)	34.8 (13.8)	26.6 (12.1)	8.2	2.0 to 14.4	0.012 *
HADS-A (0-21)	5.7 (4.8)	6.0 (4.5)	0.2	-2.1 to 1.6	0.766
HADS-D (0-21)	5.5 (4.7)	6.6 (3.8)	0.7	-2.2 to 0.7	0.271
Number of outings per week	8.5 (5.0)	8.6 (5.3)	0.1		
Number of days out the house: beyond the front door	5.3 (1.8)	6.2 (0.8)	0.9		
Number of days out the house: beyond the front gate	4.3 (2.1)	4.2 (2.2)	-0.1		

^#^Within-groups differences and confidence intervals calculated using paired t-tests (2-tailed), n = 21. Diff = Difference. 95% CI = 95% confidence interval. * Statistically significant at 0.05.

NEADL = Nottingham Extended ADL index; RNLI = Reintegration to Normal Living Index; FES-1 = Falls Efficacy Scale International; HADS = Hospital Anxiety and Depression Scale. For all measures except the FES-I and HADS, an increased total score represents improved performance or health.

^† ^Outdoor journeys were calculated by adding each 'leg' completed during an outing. For example, a person who walked to the car, travelled in a car to the shops, walked from the car into shops was recorded as having completed three outdoor journeys.

When pre-post outcomes were calculated across this small sample, within-group differences only reached statistical significance for the NEADL (7.3 points, 95% CI, 1.2 to 13.5, p = 0.022) and FES-I (8.2 points, 95% CI, 2.0 to 14.4, p = 0.012). For the key patient outcome of interest--the proportion of people with stroke who reported getting out of the house as often as they wanted--the within-group difference did not reach statistical significance (p = 0.219). The mean number of outings reported per week remained unchanged over time: 8.5 (SD 5.0) at baseline, and 8.6 (SD 5.3) at follow-up. Nor was there any significant change in the mean number of outdoor journeys or number of days out the house beyond the front door or front gate (Table 3).

#### Consenting rate for stroke patients

Almost one-half of all people with stroke referred over the 12-month period (52%) did not need or want outdoor journey sessions. These individuals did not have community participation goals, and were already getting out as often as they wanted. Further, of the 48% of stroke patients who received the outdoor journey sessions, 69% consented to provide outcome data and 31% declined.

## Discussion

To our knowledge, this is the first knowledge translation study involving community stroke rehabilitation teams. Previous studies have reported on the performance of stroke unit teams using clinical audits in hospital settings in England [25], the Netherlands [26], and Australia [27]. Until completion of this study, less was known about how community teams performed when translating evidence from stroke trials into practice.

There are three key messages from our study discussed in depth below. First, it was feasible for community teams to provide multiple outdoor journey sessions as part of their usual practice. Second, the level of behaviour change varied across teams. Third, the outdoor journey sessions led to improved outcomes for people with stroke.

### The sample

The teams appeared to be representative of non-inpatient rehabilitation stroke services in Sydney. While no database of services exists, a telephone survey was conducted informally by AM in early 2009 to any known community and outpatient service for adults with a stroke in Sydney. Results identified only two stroke-specific services in operation. Other services consisted of: three generic day hospitals/centres; at least 12 community-based transitional services for older adults recently discharged from hospital; fewer than 10 generic community-based services; and at least 15 hospital-based generic out-patient services. All of these service models were represented in our sample.

Professionals delivering the outdoor journey sessions were experienced occupational therapists and physiotherapists; all had at least five years clinical experience. Junior and recently graduated professionals are rarely employed in these positions, because of the complex caseload and clinical reasoning required.

People with stroke in both audit cohorts were similar in terms of median age (67.5 and 66.5 years respectively) and time post-discharge (median 23.5 days and 21.5 days, respectively). The median age of people with stroke in Australian hospitals is 76 years (IQR 65 to 83) [28], therefore, our audit cohorts were younger. They may have had fewer co-morbidities, however we did not record this information because of limited time. Unfortunately, we also did not record time post-stroke. In the trial by Logan and colleagues [7], people who received outdoor journeys sessions were approximately one year post-stroke, and lived at home. The 23 people in our sample had experienced their stroke more recently (they were approximately two months post-stroke), and had only been home for about three weeks.

### Feasibility and safety of the outdoor journey sessions

An important finding from this study was that therapists were able to adapt their practice over the 12-month period. It was feasible for some teams to incorporate the extra sessions into their busy programs by sharing sessions across disciplines. Role expansion and sharing were the main strategies contributing to team behaviour change, as we have reported elsewhere [20]. In the trial by Logan and colleagues [7], only occupational therapists delivered the outdoor journey sessions. However, the sessions can be delivered by physiotherapists as well as occupational therapists (Dr Pip Logan, personal communication, November 2007). In our study, some sessions were also provided by a therapy assistant. We can recommend this strategy of role sharing to other teams in future studies.

No adverse events occurred, although professionals were concerned about risk management when escorting people out into the community. Stories collected from 19 of the 23 people with stroke will be used to inform future stroke participants of the process of getting out of the house with therapy support (Barnsley, McCluskey & Middleton, What people say about travelling outdoors after a stroke: A qualitative study, submitted). Risk management strategies, such as health professionals' carrying a mobile phone and the number of a key family member, may help to alleviate concerns. People with stroke and their families can be assured that they will be well supervised, and their program upgraded safely and gradually.

Finally, it was feasible for two teams to recruit 23 people with stroke over 12 months, and consent them for outcome data collection. We had anticipated collecting data from 40 people with stroke (20 per team) in this time period, based on referrals from the previous year. However, participant numbers were about one-half of what we had anticipated. When we examined the data from one team, we found that less than 50% of their stroke caseload had outdoor mobility and community participation goals and wanted the outdoor journey intervention; of this sub-group, two thirds (69%) were recruited and provided outcome data (33% of their total stroke caseload). Therefore, about one-third of people with stroke referred to that service were eligible and consented. It is possible that team members engaged in gatekeeping, and did not recruit all eligible participants, as we observed in a previous feasibility study [29]. An independent recruiter may help to minimise this problem in future studies.

### Variations in the level of behaviour change and team functioning

Team four out-performed other teams in the pre-intervention and post-intervention medical record audits; they had higher compliance with audit criteria, provided more outdoor journey sessions per patient, and (anecdotally) engaged in more role sharing. Yet Team four employed three different occupational therapists during the year. They did not have a stable team who had worked together for many years. Team and staffing changes were experienced by all but one of the teams during the 12-month study period. However, team four had a team leader who allocated time to quality improvement, systems change, and who orientated new therapists to the project during the year.

Team functioning and characteristics have been the focus of at least two large national studies to improve outcomes post-stroke in the Netherlands [26] and United States [30]. The Dutch study recruited 14 national stroke services, paying each €15,000 to cover program costs. Teams attended four conferences on service improvement, decided on problems and bottlenecks in their service, set goals, received coaching, support, and regular feedback on their performance, as well as site visits. Team characteristics and functioning explained 40% of the variance in hospital length of stay across services. It is possible that these domains explain differences in team outcomes in our study, but we cannot be sure because team functioning was not assessed.

In the North American study, the primary aim was to test whether team training enhanced team functioning and improved outcomes post-stroke [30,31]. Training for experimental teams included financial support ($1,000 per site), a 2.5 day workshop with follow-up meetings for team leaders [32] covering topics such as team problem solving, how to use program evaluation data, and write action plans. All teams received performance feedback. Stroke patients treated by experimental teams improved by 13.6 points more than control participants on the motor items of the Functional Independence Measure. However, there was no statistically significant difference in the average length of hospital stay. Thus, there is a small but growing body of research suggesting that team coaching and training can enhance performance and improve patient outcomes. Future knowledge transfer studies should consider ways to measure, and strategies to enhance, team functioning.

### Fidelity of the intervention

One factor that we tried to maximise in this study was fidelity of the original intervention. Implementation fidelity is the degree to which programs are implemented as intended by the original developers [33,34]. Unless an evaluation is made of fidelity, service providers cannot determine if a lack of impact is due to poor implementation or problems with the program itself [33]. We wanted to ensure that what local therapists were delivering was 'true' to Logan's original randomised trial and used a number of strategies to maximise fidelity. First, the first author spent time face-to-face in 2005 and 2008 with the trialist, Dr Pip Logan, discussing the intervention. Second, a 60-page protocol was developed and provided to professionals. No such document previously existed, and can form the basis of protocols for future studies. Third, we interviewed 19 people with stroke after their sessions had concluded and eight team members about their practice, in mid 2008, prior to the second audit. We did not, however, observe sessions, and cannot be sure that what therapists recorded in the medical records reflected what they did.

### Study limitations

Our research had some limitations. First, the study was not powered to detect statistically significant differences in team or patient outcomes. We did however test the feasibility of multiple patient outcome measures to determine which instruments should be used in a future trial. Second, the absence of a control group and blinded assessor are major limitations. We do not know if the changes in team behaviour were due to the 'Out-and-About Implementation Program' or factors related to the teams and health environment at the time. Our next study, a cluster randomised controlled trial, will address this limitation by randomising teams, include control teams that receive no audit feedback, no education and do not engage in the process of barrier identification.

### Implications for practice and research

First, the current study highlights the complexity and challenges of changing practice behaviours. Small changes in practice, with large variations across teams can be expected with the first wave of implementation. Changes in the vicinity of 50% to 75% are unrealistic [18], and cannot be expected.

Second, this study has implications for routine clinical practice and education. These professionals were asked to change their practice. In some instances, change was achieved through collaboration between physiotherapists and occupational therapists, and involvement of therapy assistants. Role sharing and expansion are examples of organisational interventions [35]. A more in-depth examination of how therapists can maximise their roles may be of benefit to improve delivery of outdoor sessions to people post-stroke. Further, a process analysis alongside our proposed cluster randomised trial, examining teamwork and leadership, would also be of interest.

## Summary

Our 'Out and About Implementation Program' was feasible and safe. No adverse events were recorded when therapists delivered the outdoor journey sessions to community dwelling people with stroke. The practicalities of incorporating extra sessions into already busy work schedules can be a major impediment to practice change. Yet, multiple outdoor journey sessions were implemented by therapists; improved screening of people with stroke was conducted by team members about outings, preferred destinations, and driving. Such screening may help to raise therapists' awareness of community participation post-stroke. While 57% of people with stroke reported getting out and about as often as they liked after receiving the outdoor journey sessions, there is room for further improvement. Fidelity of the patient intervention needs to be monitored in future studies. A well-designed cluster randomised controlled trial is warranted to test the effectiveness of the implementation program and its active components: audit and feedback, barrier identification, and tailored education.

## Competing interests

The authors declare that they have no competing interests.

## Authors' contributions

The first author conceptualised and planned the study, collected and analysed the data, and drafted the manuscript. The second author advised on study design and writing of the manuscript. Both authors read and approved the final manuscript.
